# Reorganization of finger coordination patterns through motor exploration in individuals after stroke

**DOI:** 10.1186/s12984-017-0300-8

**Published:** 2017-09-11

**Authors:** Rajiv Ranganathan

**Affiliations:** Department of Kinesiology, Michigan State University, 308 W Circle Dr Rm 126, East Lansing, MI 48824 USA

**Keywords:** Stroke, Hand, Finger, Learning, Coordination, Exploration, Variability

## Abstract

**Background:**

Impairment of hand and finger function after stroke is common and affects the ability to perform activities of daily living. Even though many of these coordination deficits such as finger individuation have been well characterized, it is critical to understand how stroke survivors learn to explore and reorganize their finger coordination patterns for optimizing rehabilitation. In this study, I examine the use of a body-machine interface to assess how participants explore their movement repertoire, and how this changes with continued practice.

**Methods:**

Ten participants with chronic stroke wore a data glove and the finger joint angles were mapped on to the position of a cursor on a screen. The task of the participants was to move the cursor back and forth between two specified targets on a screen. Critically, the map between the finger movements and cursor motion was altered so that participants sometimes had to generate coordination patterns that required finger individuation. There were two phases to the experiment – an initial assessment phase on day 1, followed by a learning phase (days 2–5) where participants trained to reorganize their coordination patterns.

**Results:**

Participants showed difficulty in performing tasks which had maps that required finger individuation, and the degree to which they explored their movement repertoire was directly related to clinical tests of hand function. However, over four sessions of practice, participants were able to learn to reorganize their finger movement coordination pattern and improve their performance. Moreover, training also resulted in improvements in movement repertoire outside of the context of the specific task during free exploration.

**Conclusions:**

Stroke survivors show deficits in movement repertoire in their paretic hand, but facilitating movement exploration during training can increase the movement repertoire. This suggests that exploration may be an important element of rehabilitation to regain optimal function.

## Background

Stroke often results in impairments of upper extremity, including hand and finger function, with 75% of stroke survivors facing difficulties performing activities of daily living [1, 2]. Critically, impairments after stroke not only include muscle- and joint-specific deficits such as weakness, and changes in the kinetic and kinematic workspace of the fingers [3, 4], but also coordination deficits such as reduced independent joint control [5] and impairments in finger individuation and enslaving [6–9]. Therefore, understanding how to address these coordination deficits is critical for improving hand rehabilitation.

Typical approaches to hand rehabilitation emphasize repetition [10] and functional practice based on evidence that such experience can cause reorganization in the brain [11]. Although this has proven to be reasonably successful, functional practice (such as repetitive grasping of objects) does not specify the coordination pattern to be used when performing the tasks. As a result, because of the redundancy in the human body, there is a risk that stroke survivors may adopt atypical compensatory movements to perform tasks [12]. These compensatory movements have been mainly identified during reaching [13, 14], but there is evidence that they are also present in finger coordination patterns during grasping [15]. Although there is still debate over the role of compensatory movements in rehabilitation [16], there is at least some evidence both in animal and humans that continued use of these compensatory patterns may be detrimental to true recovery [17–19].

To address this issue, there has been a greater focus on directly facilitating the learning of new coordination patterns. Specifically, in hand rehabilitation, virtual tasks (such as playing a virtual piano) have been examined as a way to train finger individuation [20, 21]. In these protocols, individuation is encouraged by asking participants to press a particular key with a finger, while keeping other fingers stationary. A similar approach to improve hand dexterity was also adopted by developing a glove that could be used as a controller for a popular guitar-playing video game [22]. However, directly instructing desired coordination patterns to be produced becomes challenging as the number of degrees of freedom involved in the coordination pattern increase. For example, the hand has approximately 20 kinematic degrees of freedom, and providing verbal, visual or auditory feedback for simultaneously controlling all these degrees of freedom would be a major challenge. A potential solution that has been suggested is not to directly instruct the coordination pattern itself, but rather let participants explore different coordination patterns [23]. This idea of motor exploration is based on dynamical systems theory that suggests that variability and exploration may help participants escape sub-optimal pre-existing coordination patterns and potentially settle in more optimal coordination patterns [24–27]. Such exploration has been shown to be important in adapting existing movement repertoire [28], and has also been shown to be associated with faster rates of learning [29].

In order to test the hypothesis that exploration of novel coordination patterns can improve overall movement repertoire, I used a body-machine interface [30, 31] to examine how stroke survivors explore and reorganize finger coordination patterns with practice. A body-machine interface maps body movements (in this case finger movements) to the control of a real or virtual object (in this case a screen cursor), which can provide a way to elicit different coordination patterns in the context of an intuitive task. Specifically I examined: (i) how stroke survivors reorganize their finger coordination patterns, (ii) how training to explore novel coordination patterns affects their ability to reorganize their coordination pattern, and (iii) if training to explore novel coordination patterns has an effect on their overall movement repertoire. In this context, I use the term “novel” to indicate coordination patterns that require finger individuation. This assumption is motivated by the finding that stroke survivors have difficulty producing finger individuation even under explicit instruction [6, 9], and therefore it is highly likely that they would not use coordination patterns requiring finger individuation frequently in activities of daily living.

## Methods

### Participants

Participants were 10 chronic stroke survivors with mild to moderate impairment of the hand. Inclusion criteria were: a single stroke (>6 months prior to participation), and some movement of the fingers (Chedoke-McMaster > 3). Exclusion criteria were: visual or cognitive deficits that would prevent them for performing the task. Data from one participant (S05) was excluded because of technical difficulties with wearing the glove on the paretic hand. The information of the remaining 9 participants is provided in Table 1. Participants provided informed consent and procedures were approved by the Northwestern University IRB.

**Table 1:** Demographics of participants in study

Participant	Stroke type	Sex	Age (years)	Time since stroke (years)	Paretic Side	Dominant hand prior to stroke	Box and Blocks test	Jebsen-Taylor test (s)
S01	Hemorrhagic	F	60	9	R	L	16	47.46
S02	Ischemic	M	58	10	R	L	32	22.8
S03	Ischemic	M	66	4	L	R	27	23.79
S04	Ischemic	F	59	25	L	L	n/a	23.88
S06	Ischemic	M	57	3	R	R	38	19.81
S07	Hemorrhagic	F	53	9	L	R	28	26.7
S08	Ischemic	F	69	6	R	R	28	28.08
S09	Ischemic	M	71	3	L	R	41	28.78
S10	Hemorrhagic	F	55	5	R	R	46	37.99

The Box and Blocks test and the Jebsen-Taylor test were performed on the paretic side

The Jebsen-Taylor test score consisted of the cumulative time to perform 6 tasks (the writing task was not included)

One participant (S05) had to be excluded since there were difficulties wearing the Cyberglove

S04 completed the initial assessment phase, but not the training phase

### Task

**Fig. 1:**
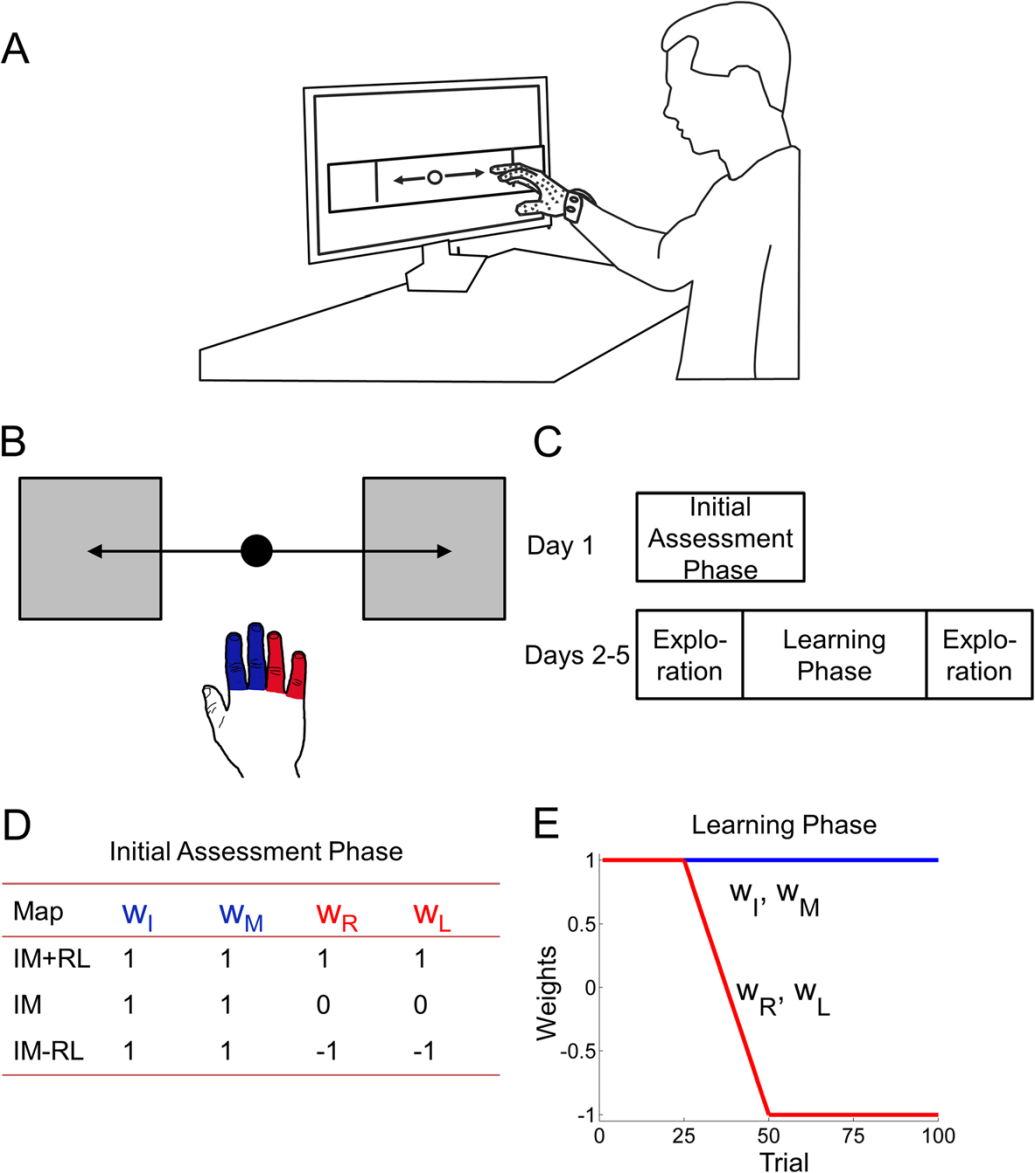
**a** Experimental setup – participants wore a data glove and moved their fingers to control a screen cursor **b** Schematic of task – participants moved a cursor between two targets using movements of four fingers (thumb excluded). **c** Experimental protocol. Participants came in for 5 total sessions – an initial assessment phase, followed by a learning phase. **d** Weighting coefficients of the index and middle (blue), and ring and little (red) fingers during the initial assessment phase, and **e** weighting coefficients during the learning phase

The task for the participants was to wear a data glove (Cyberglove, CyberGlove Systems, San Jose, CA) and control a cursor on the screen [31–33] (Fig. 1a). The data glove uses bend sensors to measure the joint kinematics of all fingers. Participants were asked to move the cursor on the screen back and forth between two targets placed at the edge of the screen [34]. Participants were asked to move both quickly and accurately to each target, stop at the target, and then make the movement to the next target (Fig. 1b). Since the focus of the experiment was on exploration, each trial was not time-limited – instead, the task was structured so that the next target would be presented only if the previous target was successfully reached. However, there was an eventual timeout criterion - in the event that a target could not be reached even after several repeated attempts, the trial was stopped when either the participant voluntarily decided to quit, or after 12 min since the previous target was captured, whichever occurred earlier.

Mapping finger movements to cursor motion:

The cursor was controlled by finger movements as follows: The data glove signals from the metacarpophalangeal (MCP) joints of four fingers (index, middle, ring and little), were mapped on to the horizontal position of the cursor on the screen using the following equation:

$$ \mathrm{x}={\mathrm{x}}_0+{\mathrm{w}}_{\mathrm{I}}{\mathrm{s}}_{\mathrm{I}}+{\mathrm{w}}_{\mathrm{M}}{\mathrm{s}}_{\mathrm{M}}+{\mathrm{w}}_{\mathrm{R}}{\mathrm{s}}_{\mathrm{R}}+{\mathrm{w}}_{\mathrm{L}}{\mathrm{s}}_{\mathrm{L}} $$

where x represents the horizontal position of the cursor on the screen, x_0_ represents an offset term designed to position the cursor on the center of the screen, w_I_ , w_M_ , w_R_ , w_L_ represent the weighting coefficients on the index, middle, ring and little fingers, and s_I_ , s_M_ , s_R_ , s_L_ represent the “normalized” signal values from the four fingers measured by the data glove. The normalized signal values were obtained by dividing the raw glove signal values from the data glove by the range of motion for that finger (i.e. the difference between the maximum and minimum glove signal values). The range of motion for each finger on each day was obtained for each individual prior to the task, where they were simply asked to flex and extend the fingers as much as possible. This normalization was done to ensure that all finger motions contributed equally to cursor motion, and that all participants would still be able to perform the task regardless of their actual range of motion (similar to normalization by maximum voluntary contraction in force production experiments).

The vector W = (w_I_ , w_M_ , w_R_ , w_L_) is referred to as the “map” (since it maps finger movements to cursor movement) and determines the set of coordination patterns that can be used to perform the task. The reason there are a set of coordination patterns (and not one unique coordination pattern) is that this mapping is redundant – i.e. it maps 4 degrees of freedom (movement of each finger) to 1 degree of freedom (horizontal position of cursor). This means that there are multiple finger postures that can be used to get the cursor to any particular location on the screen.

### Experimental Protocol

The protocol consisted of 5 separate sessions in the lab and was divided into two separate parts (Fig. 1c) – (a) an initial assessment phase (day 1), and (b) a learning phase (days 2–5) where participants learned to produce a novel coordination pattern. Participants came in twice a week for 2.5 weeks to complete the study.

#### Initial assessment phase

The map was determined by the weights on the index (I), middle (M), ring (R), and the little (L) fingers. On day 1, participants performed the cursor control task using three different maps that differed in the weighting coefficients (w_I_ , w_M_ , w_R_ , w_L_): IM + RL (1,1,1,1), IM (1,1,0,0) and IM-RL (1,1,-1,-1) (Fig. 1d). The three maps were designed to elicit different coordination patterns: the IM + RL map did not require any finger individuation and therefore was designed to be the easiest to do for stroke survivors. The IM map could also be performed without finger individuation – however, because there were only two fingers contributing to the motion, this required a larger range of motion than the IM + RL map. The IM-RL map was considered the most novel because it required some degree of individuation between the fingers in order to perform the task (without finger individuation, the cursor motion caused by the index and middle fingers would be cancelled by the opposite cursor motion due to the ring and little fingers).

The three conditions were presented in randomized order and participants performed the task both with their paretic and their non-paretic hand. The order of which hand they started with was also randomized. Each map consisted of having to reach 50 targets. In between blocks (when the maps were changed), participants were simply informed that the task difficulty could change, and that they should continue to do the task to the best of their ability, and might have to move their fingers in a different way if necessary.

#### Learning Phase

For days 2–5, participants came in twice a week to practice the novel coordination pattern in the cursor control task. To examine how participants could reorganize their coordination pattern, participants practiced a block of 100 trials where the map was changed (Fig. 1e). Participants started off with the IM + RL weighting for the first 25 trials, and then weights were gradually shifted over trials so that by trial 50, the map had shifted to the IM-RL. The map then stayed constant until trial 100. Participants were not explicitly told what the change was, other than that the task could get more difficult and that they may sometimes need to find other ways of moving their fingers to do the task. To prevent participants from anticipating the same map, I added a second block of 100 trials (either before or after the one described above) where the weights gradually changed to either IR-ML (1,-1,1,-1), or IL-MR (1,-1,-1,1) during the block. However, only the block of trials which transitioned to the IM-RL pattern was repeated every day and were analyzed for the purpose of this study.

Exploration blocks: In addition to learning the map, to examine if the training influenced their movement repertoire outside of the task, I had participants perform a “free-exploration” task where they were asked to make as many different motions with the fingers for 1 min. To ensure that I was capturing the full movement repertoire, I recorded a total of four 1-min trials - 2 before the training, and 2 after training in each session. This exploration block was a measure of the total movement repertoire. No feedback was provided to the participant during or at the end of each exploration block.

### Data analysis

#### Initial assessment phase

##### Task performance

Task performance in the cursor control task was measured by the following metrics: (i) number of targets successfully reached, (ii) movement time per target (total time at task divided by number of targets achieved), and (iii) normalized path length. (actual distance traveled between targets divided by the shortest straight line distance between the two targets). The normalized path length is a measure of the degree of control participants had over the cursor, and was used to examine if changes in movement time were simply due to changes in movement speed (in which case there should be no change in the path length).

##### Exploration of movement repertoire

To quantify the degree to which different coordination patterns were explored, I used the data from the IM-RL block (i.e. the difficult map that required finger individuation) and performed a principal components analysis (PCA) on the normalized data glove signals of the four fingers. The PCA was computed using the covariance matrix, which meant that the relative amplitudes of the finger movements was preserved. The variance accounted for (VAF) by the first principal component (PC1) was used as a measure of the movement repertoire used. A greater percentage of variance in PC1 indicates a smaller movement repertoire, and conversely, a smaller percentage indicates a larger movement repertoire. This metric is based on studies that used other dimensionality reduction techniques to quantify the complexity of the movement repertoire [35, 36].

#### Learning phase

##### Task performance

Similar to the initial assessment phase, I quantified task performance using the number of targets reached, and the movement time per target. In addition because the map changed during the course of trial, the movement time was computed in each of 10 trial blocks (each trial block consisted of 10 trials).

##### Reorganization

The reorganization in coordination patterns during the task was computed using the following measures:

(i) reorganization time – because the map changed during the course of the trial, the reorganization time was computed as the movement time in trial block 5 (i.e. trials 40–50) – the last block of the map change, when the movement times were typically the largest.
(ii) Change in PC1 – to compute a measure of how much the coordination pattern changed, I used the dot product to find the angle between the PC1 in the first trial block (i.e. 1–10), and the PC1 for every subsequent trial block. I used the maximum angle during the entire trial as a measure of the reorganization in the coordination pattern. A value close to 0 degrees indicates little reorganization from the original pattern, whereas a value close to 90 degrees indicates a large change in the coordination pattern.

##### Movement repertoire during free exploration

I computed PCA on the data from each of the 4 free exploration blocks each day. The VAF by PC1 was averaged across the four blocks to get a measure of the movement repertoire outside of the task.

### Statistical analysis

#### Initial assessment phase

Because participants were quite heterogeneous in their movement impairment (and therefore in their ability to perform the task), I used the Friedman’s test (a non-parametric test of the repeated measures ANOVA) to examine changes in movement time.

#### Learning Phase

For all dependent variables in the learning phase, comparisons were made between the first and last session (i.e. day 2 vs day 5) using the Wilcoxon signed-rank test (a non-parametric version of the paired t-test). The significance level was set at α = 0.05.

## Results

### Initial assessment phase

#### Task performance

##### Number of targets

For the paretic hand, four participants were not able to complete the full set of targets in the IM-RL map (S01: 6, S03: 13, S04: 11, and S07: 23 targets), and one participant (S01:13 targets) in the IM map. Except these, participants were able to complete the full set of 50 targets in the remaining conditions.

##### Movement time

**Fig. 2:**
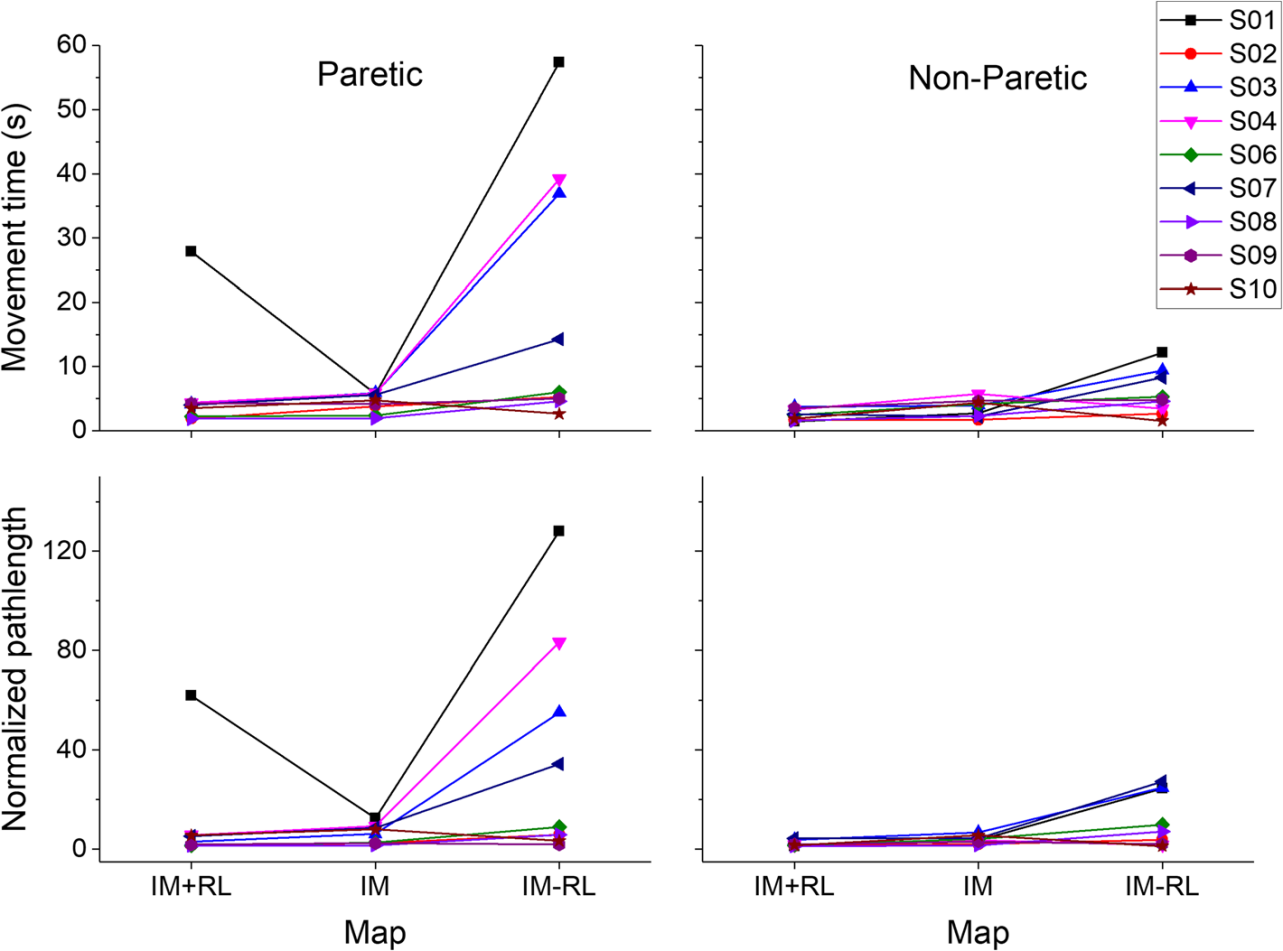
Movement time (top) and normalized path length (bottom) for the three maps in both the paretic and the non-paretic hand. The IM-RL map, which requires greater individuation between the fingers was harder to do, as indexed by both an increase in the movement time, as well as the normalized path length. S05 did not participate in the initial assessment phase

I examined the movement time for the paretic and non-paretic hand during all 3 tasks (Fig. 2, top panel). For the paretic hand, there was a significant effect of map – (Friedman’s test Z = 9.556, *p* = .008). Post hoc pairwise comparisons indicated that the movement times in the IM-RL map was significantly longer than the IM + RL map (*p* = .007). None of the other comparisons were significantly different from each other. The same pattern of results was also observed on the non-paretic hand (Friedman’s test Z = 9.556, *p* = .008, times in IM-RL longer than IM + RL). The magnitude of the difference on the non-paretic hand was much smaller than that observed on the paretic hand.

##### Path length

To examine if differences in movement time were due to differences in controlling the cursor, I also examined the path length (Fig. 2, bottom panel), which showed a similar pattern to the movement time. The correlation between the movement time and path length within each hand across all conditions was high for both the paretic hand (*r* = 0.836, *p* < .001) and the unaffected hand (*r* = 0.814, p < .001) indicating that longer movement times were strongly associated with higher path lengths (and therefore differences in movement time were not simply a matter of speed differences).

##### Movement repertoire

**Fig. 3:**
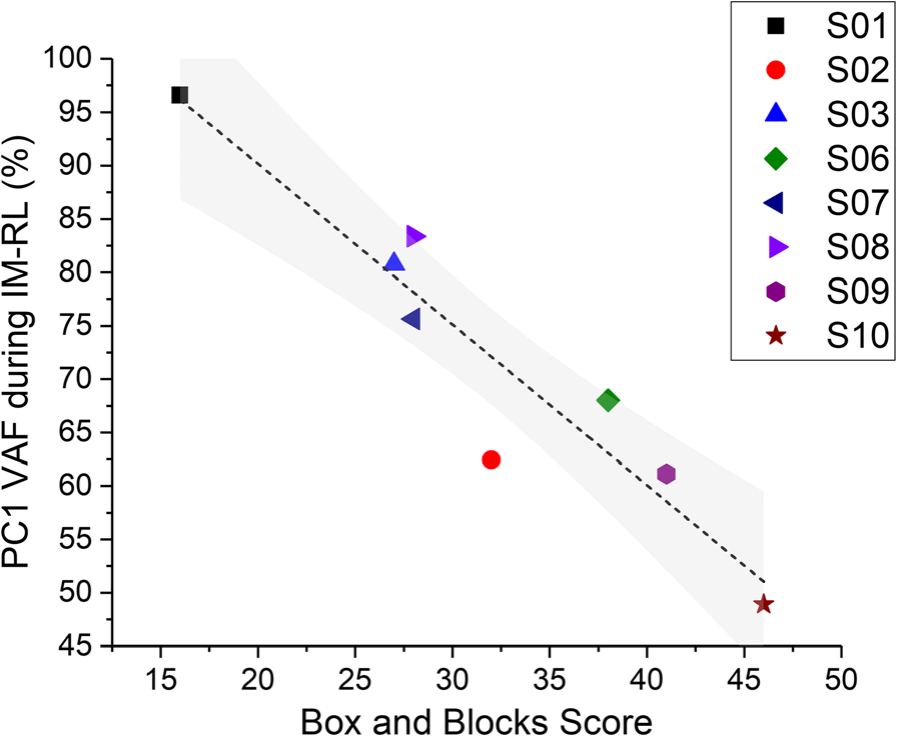
Correlation between Box and Blocks score and VAF in the IM-RL condition in the paretic hand. Participants with lesser impairment tend to generate a larger movement repertoire (as indexed by a reduced % in PC1). S04 is not included in this graph because this participant did not have a box and block score at pre-test. S05 did not participate in the initial assessment phase

When I examined the movement repertoire during the IM-RL task by using the VAF by PC1, I found a strong negative correlation between the initial level of function (as measured by the Box and Block test) and the VAF accounted for PC1 (*r* = −0.947, *p* < .001) (Fig. 3). This correlation suggests that individuals with higher box and block scores (and better function) were able to explore a greater range of coordination patterns during the difficult map.

#### Learning phase

One participant (S04) who attended the initial assessment phase was not able to participate in the learning phase of the study – as a result, the learning phase included only 8 participants.

##### Task performance

**Fig. 4:**
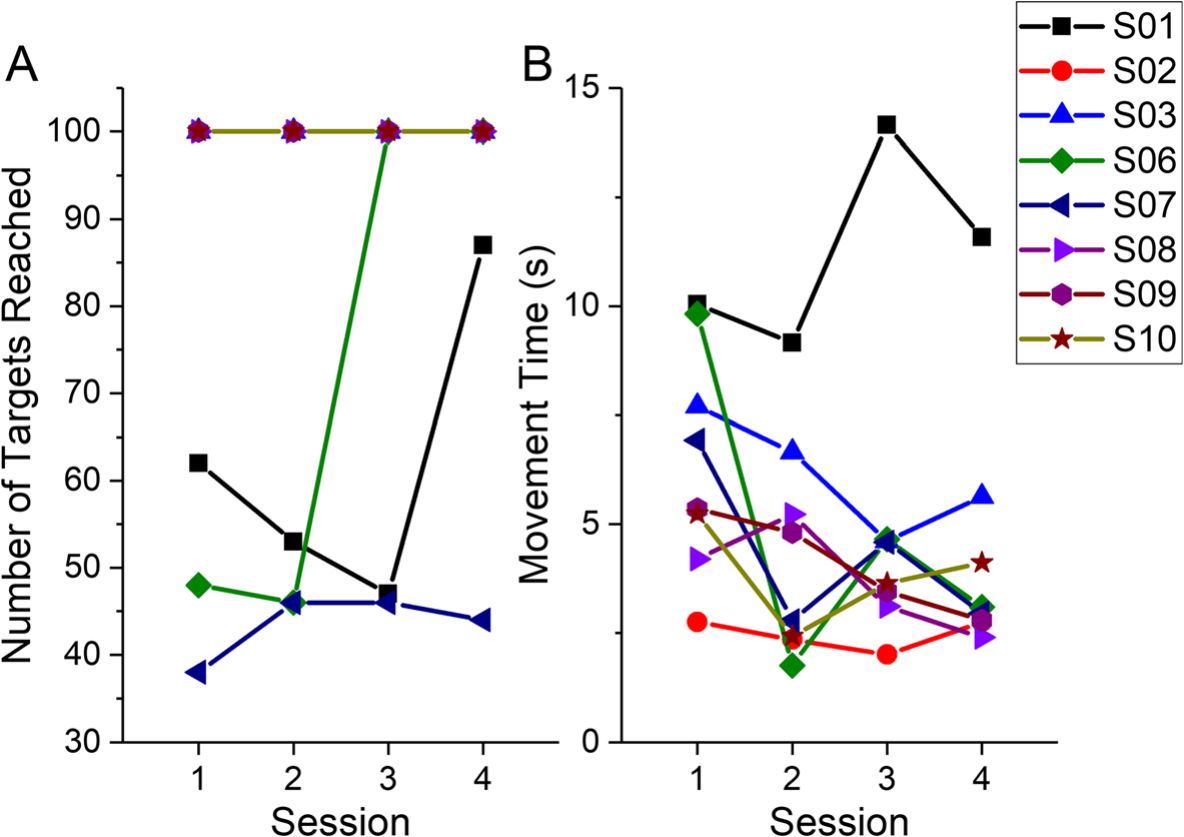
**a** Number of targets reached (out of 100), and **b** average movement time during the learning phase for all participants. S04 and S05 did not participate in the learning phase

The number of targets achieved in each of the 4 sessions, and the corresponding movement time is shown in Fig. 4. The movement time per targets decreased from the first to the last session of training (Wilcoxon Z = −2.1, *p* = .036).

##### Reorganization

**Fig. 5:**
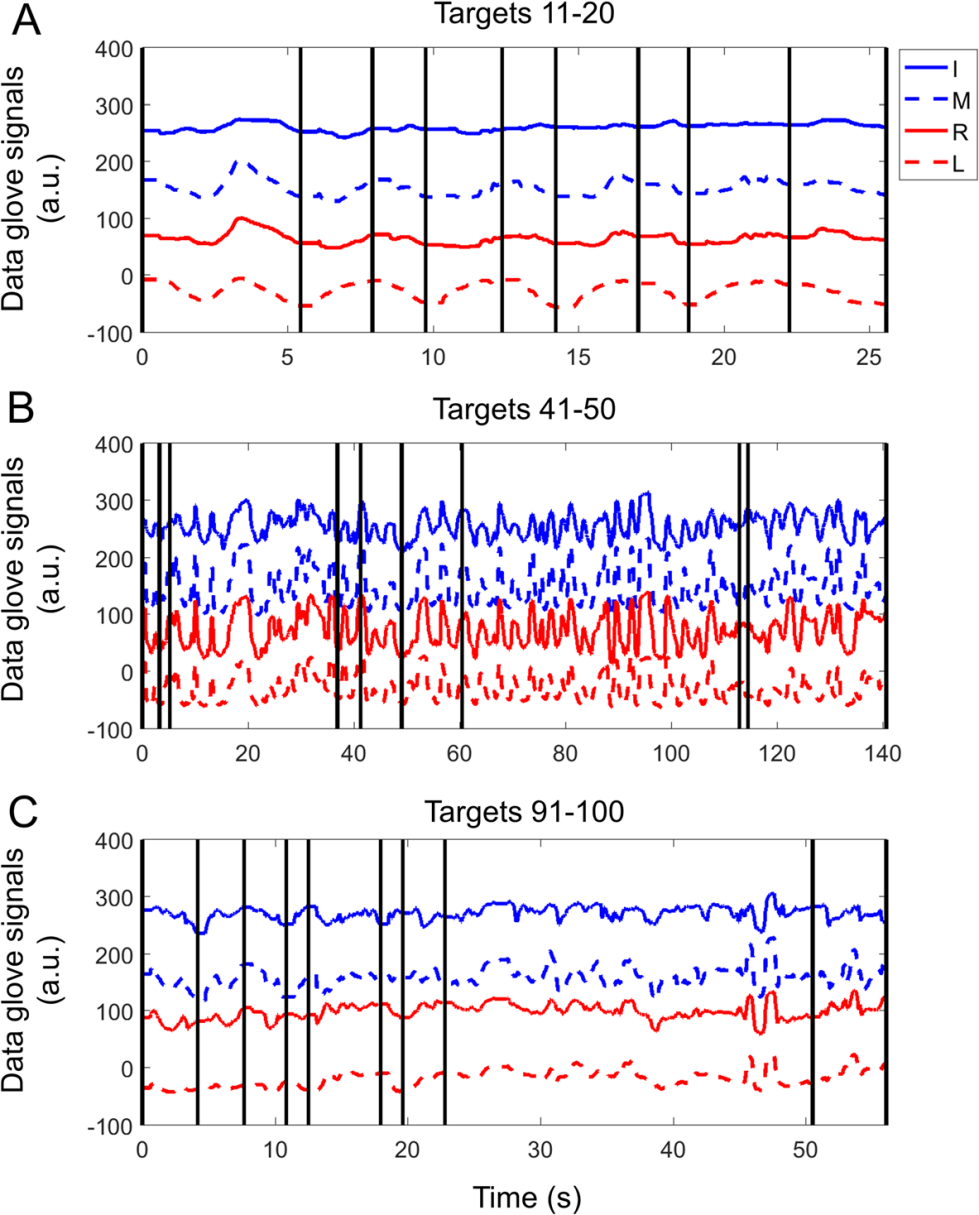
Sample data from one individual during the **a** early (targets 11–20), **b** middle (41–50) and **c** late (91–100) in the first session of the learning phase. Black vertical lines indicate the acquisition of a target. Note that the scale on the x-axis is not the same in all panels. Also the individual finger signals from the data glove (which were always between 0 and 255) are plotted with offsets for the sake of clarity

**Fig. 6:**
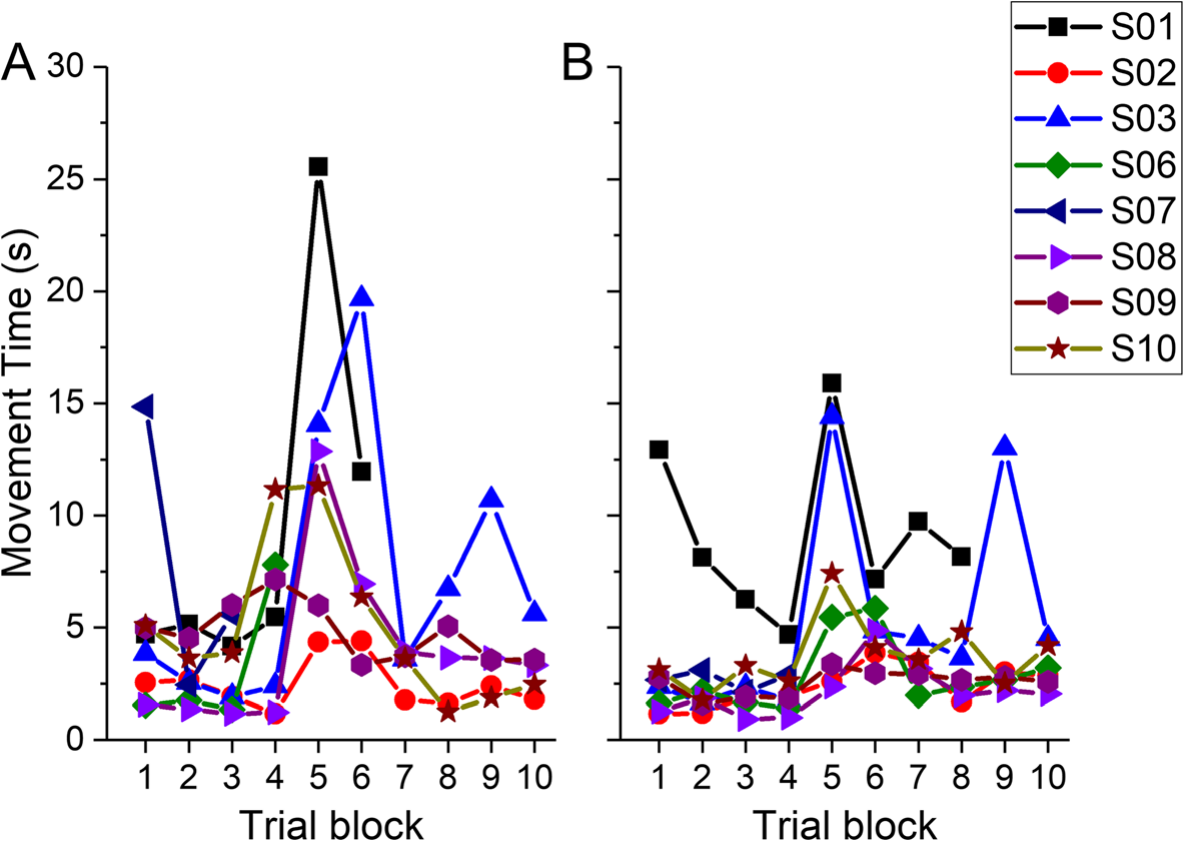
Movement time as a function of trial block in **a** first and **b** last session of the learning phase. Each trial block was the average of 10 trials. The sharp rise in times around block 5 (trials 40–50) indicate the time when participants had to reorganize their pattern to achieve the task because of the change in weighting coefficients

Sample data from one individual during the first session (Day 2) in the learning phase is shown in Fig. 5. To examine how participants were able to adapt to the change in weighting coefficients, I split the movement time across each block of 10 trials. In both the first (Fig. 6a) and last (Fig. 6b) sessions, movement times in the first three trial blocks were relatively small, there was an increase in the next two trial blocks during which participants reorganize to find a new coordination solution, and the movement time dropped again after that once a new coordination solution was found.

##### Reorganization time

**Fig. 7:**
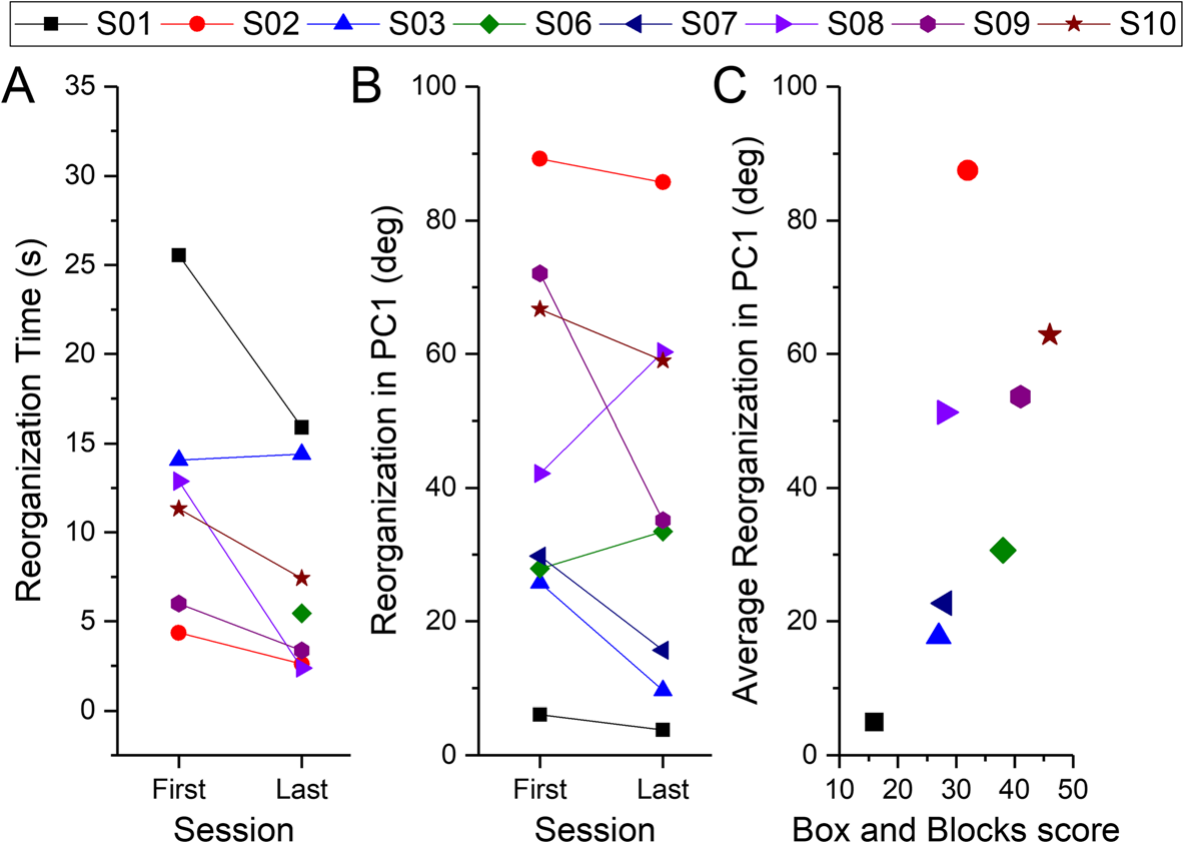
Reorganization of coordination patterns during the learning phase. **a** Reorganization time (defined as the movement time in trial block 5) in the first and last session of practice. Participants who were unable to complete trial block 5 are not shown in the figure. **b** Reorganization in PC1 (measured as the maximum deviation in angle between PC1 in the first trial block, and PC1 in any of the other trial blocks) in the first and last session of practice. **c** Scatter plot showing relationship between average reorganization in PC1 (averaged across the first and last sessions) and the Box and Blocks score at baseline

The reorganization time (defined as the movement time in trial block 5) showed a statistically significant decrease in the reorganization time from the first to the last session of training (Wilcoxon Z = −1.992, *p* = .046) (Fig. 7a).

##### Change in PC1

There was a large change in PC1 during each session from the initial trial block (mean average change = 41°) – however there was no significant change in the angle from the first to the last session (Wilcoxon Z = −1.68, *p* = .093) (Fig. 7b).

There was also a significant correlation between the average change in PC1 (averaged across the first and last sessions) and the box and block test score (Fig. 7c). Because the relationship did not seem linear, I used the Spearman’s correlation coefficient to quantify this relationship (Spearman’s ρ = 0.79, *p* = .020), indicating that participants who had greater hand function were able to reorganize their coordination pattern to a greater extent.

##### Movement repertoire

**Fig. 8:**
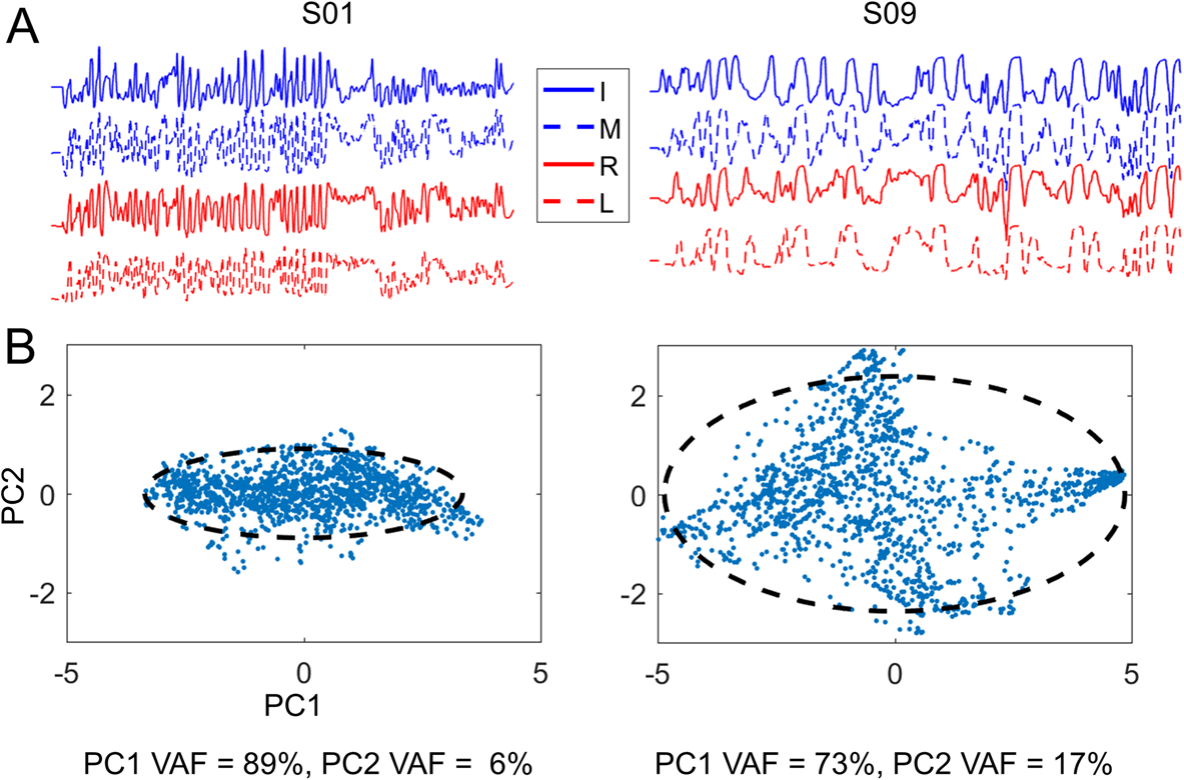
Sample data from two individuals from one exploration trial in the free exploration phase. The top panel shows the individual finger movements from the data glove, plotted as a function of time. The bottom panel shows the same data plotted in the space of the first two principal components (PC1-PC2). A 95% ellipse of this data is also shown for the sake of visualization

**Fig. 9:**
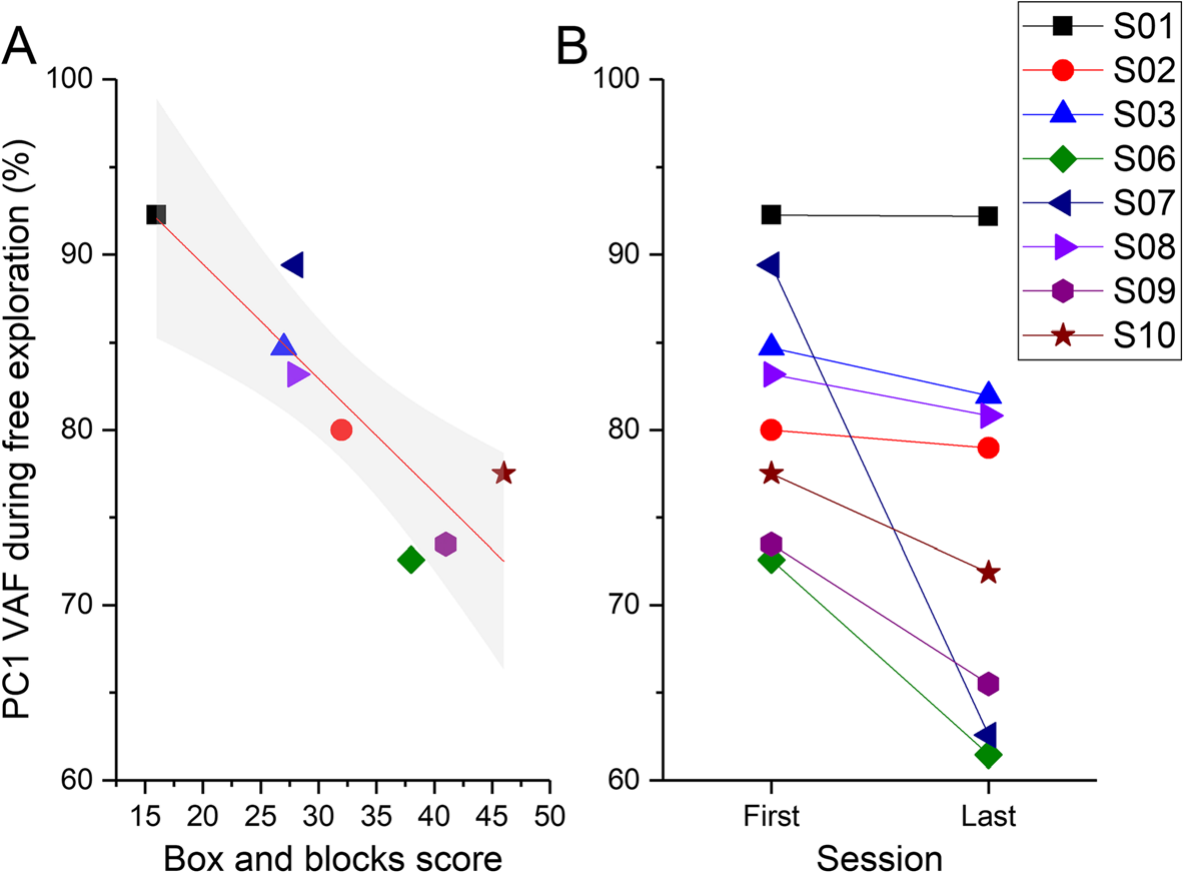
Movement repertoire in the free exploration phase. **a** Scatter plot showing relation between PC1 VAF in the first session against the Box and Blocks score. **b** Change in PC1 VAF between the first and last session of practice

Sample finger movements from two participants during the free exploration block is shown as a function of time (Fig. 8a), and plotted in the principal component space of the first two components (Fig. 8b). When examining the movement repertoire in the free exploration task, the PC1 VAF in the first session was negatively correlated with the Box and Blocks score (*r* = −.868, *p* = .005) (Fig. 9a). In addition, there was a statistically significant decrease from the first (mean VAF = 82 ± 7%) to the last session (mean VAF = 74 ± 11%) (Wilcoxon Z = −2.521, *p* = .012), indicating an increase in the movement repertoire with training (Fig. 9b).

To put the 8% drop in VAF in context, I used data from a previous study [30], where 18 young unimpaired individuals performed a very similar 1-min exploration task. The PC1 VAF in this population was approximately 66 ± 10%, which means that the 8% drop resulted in the stroke survivors on average being roughly halfway between their initial impairment (82%) and the unimpaired controls (66%).

## Discussion

The aim of this study was to examine how stroke survivors explore and reorganize finger coordination patterns. Participants used a body-machine interface that mapped their finger movements in different ways to the control of the cursor. The map between finger and cursor motion was changed without the knowledge of the participants to get participants to explore different coordination patterns to perform the task. I found the following results: (i) stroke survivors have difficulty when the mapping requires finger individuation, and the amount of exploration observed was associated with clinical tests of hand function, (ii) with 4 sessions of learning, participants were better able to reorganize their coordination patterns to perform the task requiring individuation, and (iii) this training was also associated with a modest improvement in overall movement repertoire outside of the task.

Our first finding that stroke survivors had difficulty with finger individuation is consistent with the literature [6, 9]. Movement times were larger in the IM-RL map, which required some degree of individuation between the IM and the RL fingers. Even though this result was true in both the paretic and the non-paretic hands indicating that some of this increase in movement time could be due to the non-intuitive mapping in the IM-RL map, the magnitude of increase in movement time for the IM-RL map was much larger in the paretic hand. I also found that hand function at baseline (as measured by the Box and Blocks test) correlated with the degree of exploration in the IM-RL task – i.e., the lesser the function, the less exploration there was during the task (i.e. higher the PC1 VAF). This is in line with recent evidence that that the degree of individuation is correlated to damage to the hand area in motor cortex and the corticospinal tract [37]. One important difference from earlier studies is that in the current paradigm, there was no instruction to the participants regarding the coordination pattern to be produced (because the task was redundant, there were multiple coordination solutions that could be used to reach the targets). The fact that I still found similar results suggests that reduced finger individuation may be indicative of an overall reduced movement repertoire.

The second finding was that with training, participants showed improvements in reorganizing their coordination pattern when performing the task. Participants who were unable to complete the full set of targets at the first session increased the number of targets that they were able to reach by the last session, and there was also an overall drop in the movement time. Reorganization times also decreased with practice, indicating participants were more efficient in their exploration after practice. The reorganization was also correlated with their initial hand function – this was indicated by a correlation between the change in PC1 angle and the initial Box and Blocks score. These results are consistent with evidence that stroke results in altered muscle synergies, indicating reduced movement repertoire [38] and a recent study that found that the degree of task-specific modulation in muscle activity was correlated with the level of impairment [39].

Finally, I also found changes in movement repertoire during the free exploration task after training (as quantified by the PC1 VAF). In the free exploration task, there was no cursor to control, but instead participants were simply asked to explore the repertoire of finger movements. Here, there were two observations – (i) the initial movement repertoire (the PC1 VAF) was correlated to the Box and Blocks test, indicating that initial hand function was associated with movement repertoire, and (ii) I found a decrease in VAF after 4 sessions of training, suggesting that movement repertoire improves when participants learn to explore and reorganize their finger coordination when learning the cursor control task (as seen by the changes in movement time as a function of trial block in Fig. 6).

These results point to an important, but often underappreciated role of motor exploration in rehabilitation. First, exploration can serve as an assessment tool to quantify the existing movement repertoire. For example, a recent study quantified the movement repertoire in stroke survivors using free motor exploration during a reaching task, and the results showed characteristic differences in exploration in stroke survivors that were not detected by more common measures such as range of motion [40]. Second, exploration can also serve as a rehabilitation tool by expanding the movement repertoire. Conventional task-specific training for the hand (such as precision and power grasping) only span a limited movement repertoire [41]. Repetitions of functional tasks may therefore not help participants recover the full dexterity of the hand, leading to the use of compensatory strategies. In contrast, the use of motor exploration to facilitating practice of lesser used coordination patterns may make it possible to widen the movement repertoire. However, one downside to using exploration during training is that there is no guarantee that the coordination patterns being trained are functional in any way. Therefore, one way to structure rehabilitation to get the benefit of both exploration and task-specific training may be to combine exploration as a “priming” tool to improve the repertoire and then follow up with task-specific functional training so that appropriate coordination patterns are learned.

The results also show the potential of body-machine interfaces as an important tool to reorganize coordination [30, 42]. The presence of a large number of degrees of freedom (especially in the hand) means that direct feedback on each individual degree of freedom may be impractical. By mapping this high dimensional space to the control of a low dimensional control object, body machine interfaces provide an intuitive way of eliciting different coordination patterns. This property of body-machine interfaces has typically been used for the control of assistive devices for persons with limited mobility [43–45]. However, the current results support recent evidence that these interfaces can also be used as a rehabilitation tool to improve the movement kinematics [46, 47] as well alter abnormal muscle activity [48].

There were some limitations to the study: first, this was a single group study with no control group, which makes it difficult to attribute improvements to the training protocol, and if the improvements observed in the cursor control task were simply effects of repeated exposure to the task. However, there are two reasons why I think that improvements observed are likely due to training: (i) this study was done in chronic stroke survivors who were generally several years past the stroke, making the change in the course of a few weeks less likely due to other factors, and (ii) unlike functional tests where there is clearly repeated effects of exposure, I found improvements in movement repertoire during the free exploration task, which was unrelated to the cursor control task. A second limitation was that although I was able to recruit participants with a wide range of impairment levels, all participants had to be able to wear the data glove, and have at least some volitional movement of the fingers, which resulted in a rather small sample size that potentially limits the generalizability of the findings. However, the same approach could be used in principle with more severe motor impairments using assistive robots that can aid finger movements [49].

## Conclusion

In conclusion, stroke survivors show deficits in movement repertoire that can be assessed through motor exploration. With training, participants were able to better explore novel coordination patterns outside the movement repertoire, and that also resulted in an increase in the movement repertoire. These results point out that exploration may be a critical tool to improve hand dexterity.
